# Transcriptional Start Site Coverage Analysis in Plasma Cell-Free DNA Reveals Disease Severity and Tissue Specificity of COVID-19 Patients

**DOI:** 10.3389/fgene.2021.663098

**Published:** 2021-05-28

**Authors:** Xinping Chen, Tao Wu, Lingguo Li, Yu Lin, Zhichao Ma, Jinjin Xu, Hui Li, Fanjun Cheng, Ruoyan Chen, Kun Sun, Yuxue Luo, Chen Zhang, Fang Chen, Jiao Wang, Tingyu Kuo, Xiaojuan Li, Chunyu Geng, Feng Lin, Chaojie Huang, Junjie Hu, Jianhua Yin, Ming Liu, Ye Tao, Jiye Zhang, Rijing Ou, Fang Zheng, Yan Jin, Huanming Yang, Jian Wang, Xun Xu, Shengmiao Fu, Hongyan Jiang, Xin Jin, Haiqiang Zhang

**Affiliations:** ^1^Hainan Provincial Key Laboratory of Cell and Molecular Genetic Translational Medicine, Hainan General Hospital, Hainan Hospital Affiliated to The Hainan Medical College, Haikou, China; ^2^BGI-Shenzhen, Shenzhen, China; ^3^BGI Education Center, University of Chinese Academy of Sciences, Shenzhen, China; ^4^School of Future Technology, University of Chinese Academy of Sciences, Beijing, China; ^5^Department of Hematology, Union Hospital, Tongji Medical College, Huazhong University of Science and Technology, Wuhan, China; ^6^Shenzhen Bay Laboratory, Shenzhen, China; ^7^School of Medicine, South China University of Technology, Guangzhou, China; ^8^Department of Pediatrics, Union Hospital, Tongji Medical College, Huazhong University of Science and Technology, Wuhan, China; ^9^Department of Emergency Medicine, Union Hospital, Tongji Medical College, Huazhong University of Science and Technology, Wuhan, China; ^10^James D. Watson Institute of Genome Sciences, Hangzhou, China; ^11^Guangdong Provincial Key Laboratory of Genome Read and Write, BGI-Shenzhen, Shenzhen, China

**Keywords:** plasma DNA, TSS coverage, SARS-CoV-2, tissue specificity, COVID-19 severity

## Abstract

Symptoms of coronavirus disease 2019 (COVID-19) range from asymptomatic to severe pneumonia and death. A deep understanding of the variation of biological characteristics in severe COVID-19 patients is crucial for the detection of individuals at high risk of critical condition for the clinical management of the disease. Herein, by profiling the gene expression spectrum deduced from DNA coverage in regions surrounding transcriptional start site in plasma cell-free DNA (cfDNA) of COVID-19 patients, we deciphered the altered biological processes in the severe cases and demonstrated the feasibility of cfDNA in measuring the COVID-19 progression. The up- and downregulated genes in the plasma of severe patient were found to be closely related to the biological processes and functions affected by COVID-19 progression. More importantly, with the analysis of transcriptome data of blood cells and lung cells from control group and cases with severe acute respiratory syndrome-coronavirus 2 (SARS-CoV-2) infection, we revealed that the upregulated genes were predominantly involved in the viral and antiviral activity in blood cells, reflecting the intense viral replication and the active reaction of immune system in the severe patients. Pathway analysis of downregulated genes in plasma DNA and lung cells also demonstrated the diminished adenosine triphosphate synthesis function in lung cells, which was evidenced to correlate with the severe COVID-19 symptoms, such as a cytokine storm and acute respiratory distress. Overall, this study revealed tissue involvement, provided insights into the mechanism of COVID-19 progression, and highlighted the utility of cfDNA as a noninvasive biomarker for disease severity inspections.

## Introduction

A novel coronavirus, severe acute respiratory syndrome-coronavirus 2 (SARS-CoV-2) emerged at the end of 2019 (Zhou P. et al., 2020; Zhu et al., 2020), resulting in the outbreak of the coronavirus disease 2019 (COVID-19) across the world. By April 4, 2021 (World Health Organization, 2021), more than 130 million cases were confirmed and over 2.8 million cases were dead. In a report based on nearly 72,000 COVID-19 patients from China, 14% were classified as severe, 5% were critical, and the rest 81% were considered mild (Wu and McGoogan, 2020). Clinical progression of COVID-19 varies greatly among individuals (Grasselli et al., 2020; Richardson et al., 2020; Tian et al., 2020; Wu and McGoogan, 2020; Young et al., 2020), whereas the real course of the disease is not well understood yet. In fact, the incubation period for COVID-19 ranges from 1 to 14 days, the duration of viral shedding ranges from 8 to 37 days, and the time from illness onset to death or discharge mainly ranges from 15 to 25 days (Young et al., 2020; Zhou F. et al., 2020). In addition, the case-mortality rate was found to be correlated with age and preexisting comorbidities such as cardiovascular disease, diabetes, and hypertension. However, reported deaths still contain high numbers of teenagers and cases without comorbidities (Grasselli et al., 2020; Richardson et al., 2020; Tian et al., 2020; Wu and McGoogan, 2020; Young et al., 2020). Laboratory records such as low lymphocyte counts, high C-reactive protein or D-dimer levels, and secondary bacterial infections could not provide insights into the actual process of death (Phua et al., 2020; Vincent and Taccone, 2020). Hence, systematical understanding of clinical course of COVID-19 and classification/prediction of severe cases precisely at early stage is essential for the management of the disease.

Cell-free DNA (cfDNA) in plasma comprises short, naturally fragmented molecules that preserve valuable information related to gene expression and nucleosome footprint related to its tissues-of-origin (Sun et al., 2015, 2019; Snyder et al., 2016; Ulz et al., 2016; Thierry and Roch, 2020). Numerous studies reported that cfDNA concentration, size profiles, and coverage patterns around promoters are associated with various diseases, making cfDNA an intensively investigated biomarker for clinical use in various fields including oncology, noninvasive prenatal diagnosis, organ transplantation, autoimmune diseases, trauma, myocardial infarction, and diabetes (Sun et al., 2015, 2019; Snyder et al., 2016; Thierry and Roch, 2020). Circulating cfDNA mostly originates from dead cells through apoptosis, necrosis, and NETosis (Barnes et al., 2020; Thierry and Roch, 2020; Zuo et al., 2020) and was found to be potential drivers and therapeutic targets of COVID-19 (Barnes et al., 2020; Thierry and Roch, 2020). By using genome-wide methylation profiling of cfDNA in plasma, Cheng et al. (2020) revealed the injury of lung and liver, as well as the involvement of red blood cell progenitors associated with severe COVID-19, showing the potential to predict the COVID-19 severity by plasma DNA. However, this methylation-based approach requires more plasma volume, complicated bisulfite treatment during library preparation, and is high cost, which may not suit routing screen and monitoring. Hence, to further explore the clinical utility of cfDNA in COVID-19, we conducted a systematical analysis of whole genome sequencing (WGS) data on cfDNA from mild and severe cases in time series and proposed a novel algorithm to deduce the mixed expression profile in plasma DNA. In this work, we discovered significantly different signals between mild and severe cases. These signals indicate potential genes and pathways involved in disease course and severity, demonstrating high value in patient monitoring. Our functional analysis of cfDNA further uncovered the altered biological activities in lung and blood cells of severe patients. These significant findings proved the clinical utility of cfDNA as a promising noninvasive biomarker for disease severity inspections of COVID-19.

## Materials and Methods

### Data Collection

For HN sample set, a total of 10 plasma samples were collected from two patients with COVID-19 at four time points and two healthy controls. Patients with COVID-19 were recruited from the Hainan General Hospital, Hainan, China. Healthy subjects were recruited as controls. For WH sample set, all mild and severe patients were recruited from the Union Hospital, Tongji Medical College, Huazhong University of Science and Technology, Wuhan, China. The study was approved by the Medical ethics committee of Union Hospital, Tongji Medical College, Huazhong University of Science and Technology, and the Institute Review Board of BGI, with written informed consent.

### Library Preparation

Peripheral blood was stored using EDTA anticoagulant-coated tubes. The blood sample pretreatment and DNA extraction were proceeded at a Biosafety Level 2 (BSL-2) laboratory to ensure the appropriate biosafety practices (WHO, 2020). All samples were centrifuged at low speed (3,000 rpm) for 10 min at 4°C within 6 h after collection. The supernatant was centrifuged at high speed (14,000 rpm) for 10 min at 4°C. Then, the plasma was placed at 56°C water bath for 30 min. Circulating cfDNA was extracted from 200 μl plasma using MagPure Circulating DNA Mini KF Kit (MD5432-02) following the manufacturer’s guide. The cfDNA was eluted by 200 μl TE buffer for quality check and 40 μl for the rest. For cfDNA library construction, the extracted cfDNA was processed to library using MGIEasy Cell-free DNA Library Prep kit (MGI, cat. No.: AA00226).

### Sequencing of DNA Libraries and Data Alignment

Pair-end sequencing was performed on the libraries of HN and WH sample sets by MGI DIPSEQ platform. One hundred nucleotides were sequenced for each sequencing read.

For upstream data processing, first, SOAPnuke (version 1.5.0) (Chen et al., 2018) was used to trim the sequencing adapters from raw reads and filter reads with low quality or high ratio of “N.” Second, BWA (version 0.7.17-r1188) (Li and Durbin, 2010) was employed to align the clean reads to the human reference genome (GRCh38/hg38). The above steps were performed by Sentieon (Kendig et al., 2019), an integrated platform for processing genomic data including quality control and alignment.

### Comparison of Overall Coverage in TSS Regions Among Cases and Controls in HN Sample Set

**FIGURE 1:**
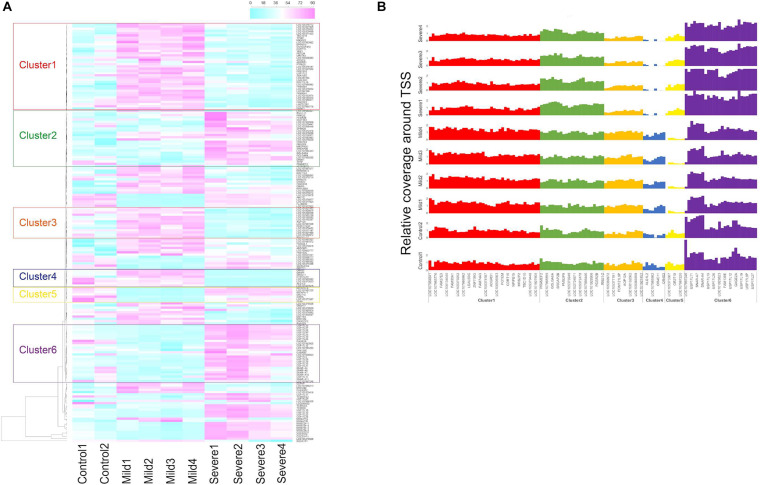
Gene clustering based on transcriptional start site (TSS) coverage in controls, mild, and severe coronavirus disease 2019 (COVID-19) cases. **(A)** Gene clustering based on relative TSS depth in plasma samples of two control individuals and COVID-19 cases collected at hospitalization days 11 (Mild1), 17 (Mild2), 19 (Mild3), and 22 (Mild4) for mild case and days 16 (Severe1), 19 (Severe2), 25 (Severe3), and 29 (Severe4) for severe case. Color scale represents average coverage around TSSs of each gene weighted by average whole-genome sequencing depth from plasma cell-free DNA (cfDNA). **(B)** Relative coverage around TSS of genes from the six identified clusters in control, mild, and severe cases.

First, for each gene, average sequencing depth within 2 and 1 kb region around transcription start sites (2- and 1-kb TSSs) were calculated, respectively, by depth of coverage package from GATK (McKenna et al., 2010). The relative coverage around TSS was the above depth normalized by average depth of WGS data from each sample. Second, for each gene, we calculated *S*_*i*_ = *D*_*maxi*_ - *D*_*mini*_ representing the difference between the highest and lowest depth among group *i* within 2-kb TSSs. Genes with *S*_*controls*_ > *S*_*cases*_ were filtered out, and the remaining genes were ranked by *S*_*cases*_. Gene clustering analysis was performed for the top 1% ranked genes based on the 2-kb-TSS coverage by the heatmap package from R version 3.5.1, and clusters of genes were selected from dendrogram output by heatmap package. Results based on 2-kb-TSS regions are presented in Figure 1.

### Analysis of Genes With Differentially Covered TSS Regions

**FIGURE 2:**
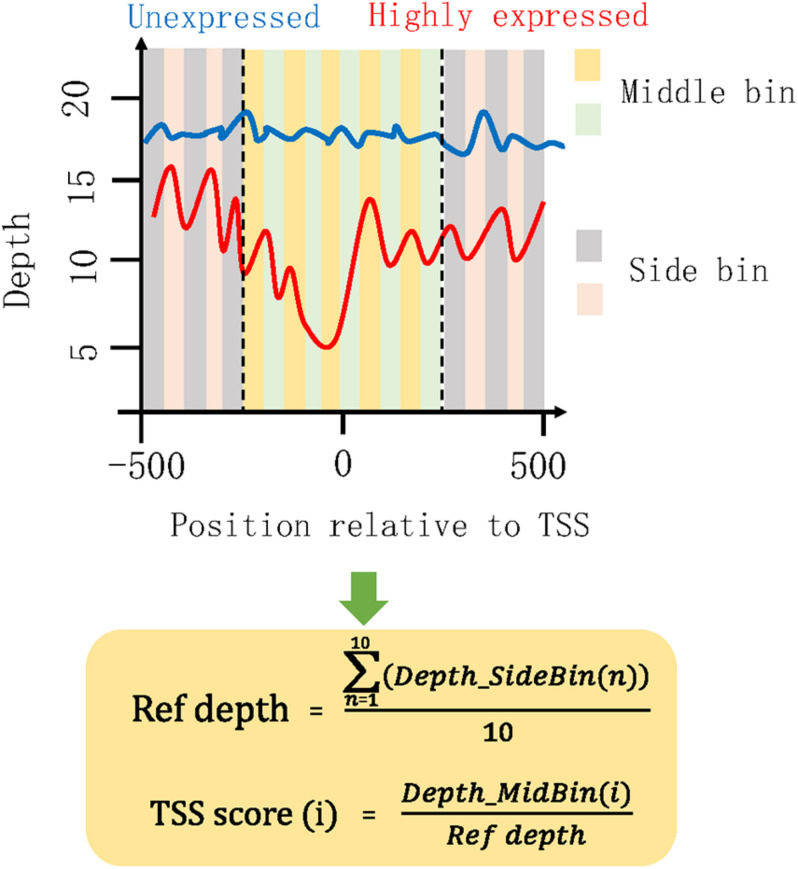
Illustration of algorithm for measuring TSS coverage profile. The coverage of 1,000 bp region surrounding TSS was investigated. Then, the 1-kb region was separated into 20 small bins with a size of 50 bp. We defined the 10 bins in the middle of this 1-kb region as the middle bins and the outward 10 bins on both sides as the side bins. *Depth_SideBin(n)* represents the depth for the *n*th side bin. The average depth (*Ref depth*) in all side bins was calculated and used for the normalization of middle bins. Here, we proposed a “TSS score” as the normalized coverage for each middle bin. *i* represent the *i*th middle bin. All the 10 TSS scores of middle bins were used to measure the chromatin states in this TSS region. A list of high TSS scores would represent a high coverage around the TSS, indicating that some specific proteins or nucleosomes were presented in this region and protected the cfDNA here from digestion. Under this circumstance, the occupied TSS regions would hamper the binding of transcription factors and result in a low expression level of this gene. Conversely, the low TSS scores were associated with high expression level of this gene.

To mine the genes with differentially covered TSS regions between mild and severe groups, we proposed a concept of TSS score to measure and compare the TSS coverage profile of each gene in the plasma DNA of healthy subjects and COVID-19 patients (Figure 2). The TSS scores of plasma samples collected from different timepoints were averaged in each middle bin for the mild and severe patients in the HN sample set. The TSS scores of plasma samples were also averaged in each middle bin for mild and severe groups in the WH sample set. Genes with significantly different TSS scores between severe and mild cases were identified as differential genes. Those genes showed significant difference in two control subjects by the same analysis were filtered from the obtained gene list.

### Analysis of Transcriptome Data Downloaded From Public Databases

To explore the potential tissues involved in the specific expression pattern in plasma, we analyzed the RNA-Seq datasets related to SARS-CoV-2 downloaded from the GEO database (Barrett et al., 2013). One is the buffy coat cells of ICU patients with and without SARS-CoV-2 infection (Gill et al., 2020) (GSE154998), and the other is the lung A549 cell with and without SARS-CoV-2 infection treatment (Blanco-Melo et al., 2020) (GSE147507).

For the transcriptome data of lung cells, we compared the gene expression patterns and identified 4,016 and 3,048 genes with significantly up- and downregulated expression levels in case group compared with controls, respectively. Among these differential genes, 353 upregulated and 96 downregulated genes were overlapped with the significant genes showing consistent pattern in plasma.

For the analysis of expression patterns in blood cells, we directly downloaded a list of differentially expressed genes described in a published research (Gill et al., 2020), which identified 254 and 1,057 genes as significantly up- and downregulated in case group, respectively. Among these genes, 16 and 37 showed consistently altered expression patterns in the plasma of our severe cases.

### Statistical Analysis

For both the HN and WH sample sets, to identify genes with significantly increased and decreased TSS coverage in severe patient, we performed the one-tail Wilcoxon signed-rank test for the 10 TSS scores of middle bins in each TSS region between mild and severe cases. A *p* value of <0.05 was considered statistically significant. For the transcriptome data of lung cells, the R package DESeq2 (Love et al., 2014) was employed to analyze the expression matrix. A significance level of adjusted *p* value of 0.05 was adopted to identify the differentially expressed genes in severe patients.

## Results

### Differential Coverage in TSS Regions Among Control and COVID-19 Patients

Four subjects, including two male COVID-19 patients (one mild and one severe) and two healthy controls (one male and one female), were recruited in this study (HN sample set). For the COVID-19 patients, peripheral blood was collected at various time points within 29 days of hospitalization; plasma cfDNA was extracted and sequenced to a median of 14.1× (range: 5.1×–37.7×) human haploid genome coverage at each time point (Supplementary Figure 1). The sequencing depths around TSS regions were explored and normalized by the average depth of whole genome as relative TSS coverage in the plasma samples of control subjects, mild and severe COVID-19 patients (“Materials and Methods” section). To compare the coverage patterns of TSS regions in cases and controls, we performed gene set enrichment analysis (GSEA) on the selected genes whose TSS regions showed large difference in coverage for all plasma samples (Figure 1). We identified six gene clusters in which the TSS coverage patterns between mild and severe cases were significantly different, suggesting the fragmentation patterns in the TSS regions of these genes were changed due to the alteration of chromatin states of these areas in severe cases (Figures 1A,B). Notably, the average coverage around gene promoters from clusters 2 and 6 decreased along hospitalization timeline for the severe cases (suggesting upregulation of these genes), while such pattern did not exist in mild cases (Figure 1A), indicating that the genes involved in disease course could be different in mild and severe cases.

### Identification of Genes With Significantly Altered TSS Coverage Profile in Severe Cases

**FIGURE 3:**
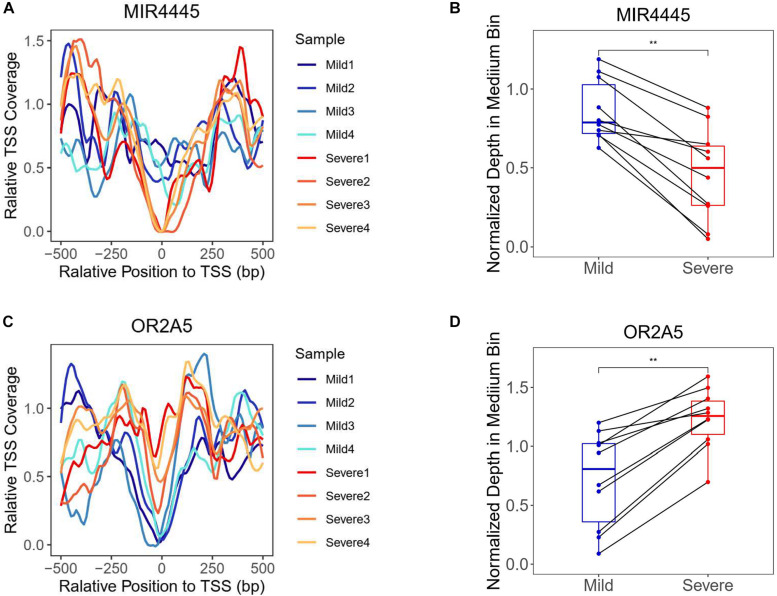
Transcriptional start site coverage profiles of two representative genes showing significantly increased and decreased trends in severe cases compared with mild cases. **(A)** Relative TSS coverage of gene MIR4445 in plasma samples of mild and severe patients. **(B)** Boxplot of TSS scores of gene MIR4445 in the plasma samples of mild and severe patients. Points of the same middle bin in two patients were linked by the black line. **Adjusted *P* value is below 0.01. **(C)** Relative TSS coverage of gene OR2A5 in plasma samples of mild and severe patients. **(D)** Boxplot of TSS scores of gene OR2A5 in the plasma samples of mild and severe patients.

The chromatin states around TSS have been found to be associated with transcription activity (Schones et al., 2008; Venkatesh and Workman, 2015). A reduction of nucleosome occupancy in TSS regions is always linked to the active transcription. In contrast, the inactive promoters are likely to exhibit the phasing of nucleosome in TSS region. Previous study has also demonstrated the feasibility of inferring expression status based on the cfDNA coverage in TSS regions of corresponding genes (Ulz et al., 2016). To distinguish the highly and lowly expressed genes in severe cases compared with mild cases, we developed an algorithm for the measurement of TSS coverage profile on the basis of the relative depth in the 500-bp region around TSS (Figure 2). Finally, we identified 988 and 2,383 genes that showed significantly higher and lower TSS coverage in severe patient (Supplementary Table 1). In Figure 3, we presented two genes showing representatively differentiated TSS coverage. In severe cases, for gene MIR4445, the relative TSS coverages were distinctly lower around TSS (Figure 3A) and the TSS scores (normalized depth of middle bins) were also significantly declined (*P* value: 0.002) (Figure 3B), which suggested that this gene expression was enhanced in the severe patients. In contrast, for gene OR2A5, the TSS coverage and TSS scores were elevated in severe cases (*P* value: 0.002) (Figures 3C,D), indicating a decreased expression level in this severe patient. These observations demonstrated that using this algorithm based on the normalized depth in the middle bins allowed us to differentiate the TSS coverage profiles and deduce expression patterns in mild and severe cases.

### Enriched Pathways of Genes With Significantly Altered TSS Coverage in Severe Cases

**FIGURE 4:**
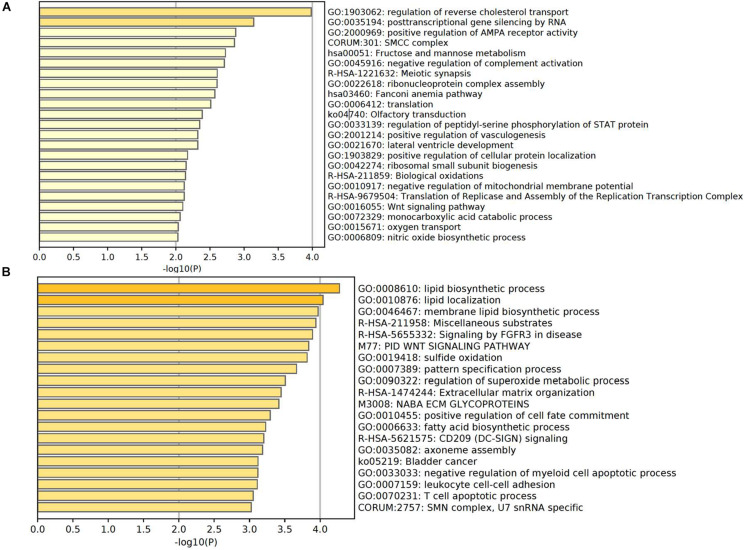
Enriched pathways of genes with significantly higher in panel **(A)** and lower in panel **(B)** coverage of TSS region in severe patient compared with mild patient.

Based on the principle that the inactive promoters with occupied TSS regions are able to prevent the plasma DNA from digestion and lead to the observation of high DNA coverages in these genomic regions, the significantly altered genes with declined and elevated TSS coverage in severe patients were regarded as the genes with up- and downregulated expression. To further investigate the functions of those genes, we applied the Metascape (Zhou et al., 2019) to perform the comprehensive pathway enrichment analysis including Gene Ontology (GO) biological processes, Kyoto Encyclopedia of Genes and Genomes (KEGG) pathway, Canonical pathways, Reactome gene sets, and CORUM on the up- and downregulated gene sets in severe cases, respectively. The enrichment results of dysregulated genes revealed that the downregulated genes were predominantly enriched in the pathways of biological processes affected by COVID-19 infection, including the regulation of reverse cholesterol transport, posttranscriptional gene silencing by RNA, positive regulation of AMPA receptor activity, fructose and mannose metabolism, negative regulation of complement activation, olfactory transduction, regulation of peptidyl-serine phosphorylation of STAT protein, etc. (Figure 4A). Most of the pathways were evidenced to be associated with the progression of COVID-19 disease. For example, the immune-mediated inflammatory dyslipoproteinemia caused by the “cytokine storm” underlying COVID-19 would lead to a low HDL-C level, whose function was to promote reverse cholesterol transport (RCT) from the periphery to the liver (Sorokin et al., 2020). The fructose and mannose metabolism pathway was reported to be one of the differentiating metabolites that are significantly enriched in symptomatic COVID-19 groups compared with the healthy group (Wu et al., 2020), and the downregulation of this pathway was consistently observed in the transcriptomes from samples of bronchoalveolar lavage fluid in COVID-19 patient (Gardinassi et al., 2020). Gralinski et al. (2018) also discovered that the complement activation regulated a systemic proinflammatory response to SARS-CoV infection which made the complement system a critical host mediator of SARS-CoV-induced disease. In addition, disturbances in smell have been commonly reported as the main neurological symptom of COVID-19 disease (Cooper et al., 2020; Galougahi et al., 2020; Lechien et al., 2020; Parma et al., 2020) and the olfactory loss was proved to be more effective in the prediction of COVID-19 infection in a recent study based on two million participants (Menni et al., 2020). GSEA on upregulated genes also uncovered a series of pathways involved in COVID-19 disease-related biological responses (Figure 4B). For example, the top three significantly enriched pathways were related to lipid biosynthetic process. According to previous studies, the host lipid biogenesis pathways were crucial in controlling virus replication because lipids were direct receptors or entry co-factors for all kinds of viruses at the cell surface or the endosomes (Taube et al., 2010; Chukkapalli et al., 2012; Bagam et al., 2017; Abu-Farha et al., 2020). Lipids were also involved in the regulation of cellular distribution of viral proteins, the formation of viral replication complex, and the energy required for viral replication (Diamond et al., 2010; Hsu et al., 2010; Mankouri et al., 2010; Nagy et al., 2016). The upregulation of these pathways suggested the active COVID-19 progression and the corresponding biological responses in this severe patient. Besides the lipid-related pathways, we also observed the significant enrichment of upregulated genes in CD209 (DC-SIGN) signaling pathway. In a previous study, the CD209L/L-SIGN and the related protein CD209/DC-SIGN were identified as receptors in mediating SARS-CoV-2 entry into human cells, which further indicates the active viral infections in this severe patient. Another notable pathway is the T cell apoptotic process, which has been reported to be enhanced in severe patient compared with mild cases (Cizmecioglu et al., 2020).

### TSS Coverage Profile in Plasma Reveals the Tissue-Specific Expression Pattern

**FIGURE 5:**
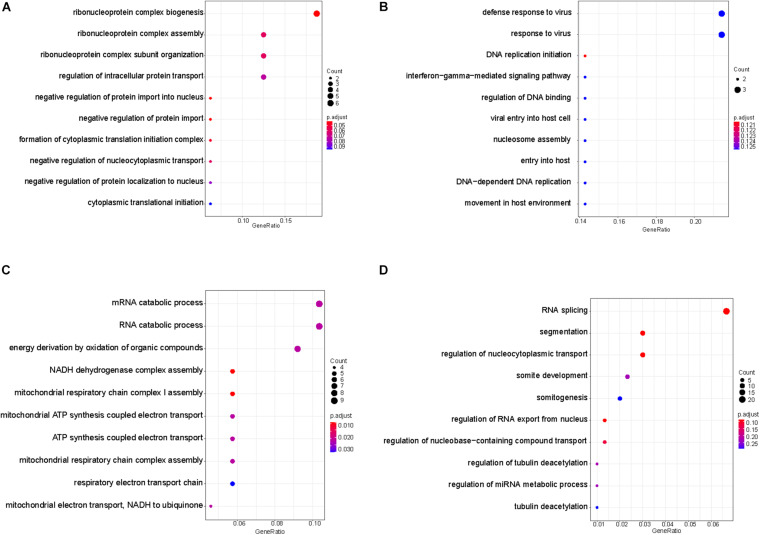
Enriched pathways of genes with consistent alteration of gene expressions in both plasma and tissues. Enriched pathways of genes with consistent down- in panel **(A)** and upregulated pattern in panel **(B)** in plasma of severe patient and blood cells of COVID-19 patients. Enriched pathways of genes with consistent down- in panel **(C)** and upregulated pattern in panel **(D)** in plasma of severe patient and lung cells with SARS-CoV-2 infection.

As plasma contains cfDNA released from multiple tissues of the body, we wonder whether the TSS coverage profile in plasma could reflect the tissue specificity. In plasma, the blood cells were reported to be the predominant contributor in the plasma DNA pool (Sun et al., 2018). In addition, as the lung is the primary target of the COVID-19 virus infection, lung-released cfDNA has been observed to be elevated in the plasma of COVID-19 patients (Cheng et al., 2020), indicating the injury occurred in the lung tissue. To dissect the tissue specificity of blood cells and lung cells involved in the mixed expression profiles in the plasma of severe patient with COVID-19, we downloaded the public transcriptome data of buffy coat cells of ICU patients with and without SARS-COV-2 infection (Gill et al., 2020) and lung A549 cells treated and not treated by SARS-COV-2 infection (Blanco-Melo et al., 2020). The genes with significantly increased and decreased expression levels in blood cells and lung cells with COVID-19 infection were compared with the significantly altered genes deduced from the TSS coverage in cfDNA of our severe cases compared with mild cases. As shown in Supplementary Figure 2A, among the upregulated genes identified in severe cases, 353 and 16 genes showed consistently elevated expression levels in infected lung cells and blood cells of COVID-19 patient (Supplementary Table 2). Meanwhile, among the downregulated genes identified in severe cases, 96 and 37 genes showed consistently declined expression levels in infected lung cells and blood cells of COVID-19 patient (Supplementary Figure 2B and Supplementary Table 2). GO analysis were further performed by clusterProfile (Yu et al., 2012) on these overlapped genes in biology process level to investigate the biological function in relation to the progression of COVID-19 in severe case. Interestingly, we found that the downregulated genes in blood cells were mostly enriched in the pathways related to the biological process of ribonucleoprotein complex and the regulation of protein transport (Figure 5A). Ribonucleoprotein complex has been revealed as the major cell processes of the SARS-CoV-2-host interacting proteins (Gordon et al., 2020), and the protein transport pathway was also reported to significantly enrich host cell proteins that comprise the coronaviral replication/transcription complex microenvironment (V’kovski et al., 2019). Moreover, through the pathway analysis on the consistently upregulated genes in blood cells, we observed that the altered genes were predominately enriched in the pathways related to the defense response to virus and viral infection in host cells (Figure 5B), suggesting that both the antiviral and viral activities were active in the blood cells of severe patient compared with the mild patient. This finding also provided a feasibility of measuring the severity of COVID-19 only from the TSS regions of plasma DNA in patients. In the meantime, we found that the genes downregulated in both plasma and lung cells were mostly located in the mRNA and RNA metabolic pathways, as well as the adenosine triphosphate (ATP) synthesis during cellular respiration (Figure 5C), which reflected the severe injury of lung tissue during the SARS-CoV-2 infection. Importantly, it has been reported that people with low ATP and low energy reserves were more likely to develop severe COVID-19 symptoms (Patel and Sriram, 2009; van Kempen and Deixler, 2020), such as a cytokine storm and ARD since the depletion of intracellular ATP may cause cell death by necrosis and membrane instability, leading to the release of ATP into extracellular space (Le et al., 2019), which would over-activate the immune system (Iyer et al., 2009) and result in these severe consequences (Trautmann, 2009; Nomura et al., 2015; Kouhpayeh et al., 2020). Whereas, the pathways enriching the upregulated genes in lung cells were mainly involved in RNA splicing, segmentation, regulation of nucleocytoplasmic transport, somite development, and regulation of RNA export from nucleus (Figure 5D).

As there are only one mild and one severe patient included in the HN sample set, to consolidate our findings in the plasma of severe patient, we further collected 10 plasma samples from five mild and five severe patients with COVID-19 from another hospital (WH sample set). Plasma DNA of these samples was sequenced to similar depth with a median of 15.8× (range: 10.4×–24.8×). Among the upregulated genes detected in the plasma of severe patients, we identified 989 and 69 genes showing consistently elevated expression levels in SARS-CoV-2-infected lung cells and blood cells of COVID-19 patient. Among the downregulated genes identified in severe cases, we identified 820 and 299 genes showing consistently declined expression levels in SARS-CoV-2-infected lung cells and blood cells of COVID-19 patient (Supplementary Figure 3 and Supplementary Tables 1, 2). More importantly, the similar enriched pathways of these differential genes were clearly observed in this new dataset. For example, the upregulated genes in the blood cells of COVID-19 patients and in the plasma of severe cases were also predominantly involved in the pathways of virus defense and virus response. Meanwhile, the genes downregulated in both the plasma of severe patients and infected lung cells were also enriched in pathways of ATP metabolic and energy derivations (Supplementary Figure 4). These results vastly enhanced our findings in the plasma of severe patients, indicating that the tissue specificity and disease severity were able to be steadily measured through the analysis of plasma DNA.

### Microbial and Mitochondrial cfDNA

**FIGURE 6:**
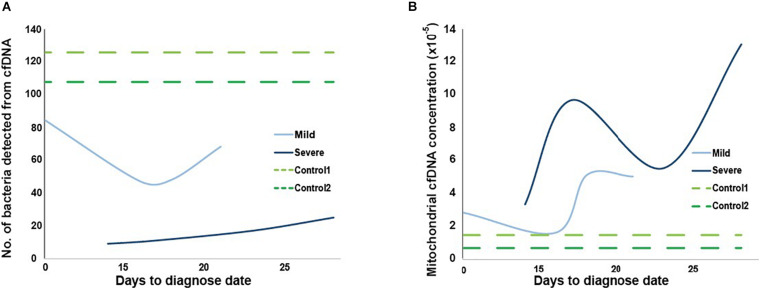
Microbial and mitochondrial cfDNA from two controls and two mild and severe cases. Number of bacteria types detected in panel **(A)** and mitochondrial cfDNA concentration in panel **(B)** from cfDNA of plasma collected at four time points for mild (light blue) and severe (dark blue) cases, and at once for the two cases (green, time for dotted lines are invented only for comparison).

Besides autosomal cfDNA from cases and controls, infection of microbiomes in plasma and mitochondrial cfDNA concentration were also investigated in the HN sample set (Figure 6). Consistent with the RNA-virus nature of SARS-CoV-2, we did not find any viral DNA of SARS-CoV-2 in the cfDNA sequencing data. Total counts of bacteria detected in the plasma from COVID-19 patients were lower than that from controls (Figure 6A), which could be explained by medication of interferons and antibiotics for these patients. Notably, a novel virus infected in plasma collected at third and fourth time points of the severe case was human betaherpesvirus 5 (Supplementary Table 3), which might cause pneumonia, colitis, or encephalitis in immunocompromised people (Taylor, 2003).

Overall, mitochondrial cfDNA concentrations of plasma from controls were lower than cases, while the severe case had a higher concentration than mild case (Figure 6B). Notably, distribution of mitochondrial concentration for severe case showed a clear “S” shape along time series, which was matched with records of hematocrit and hemoglobin at corresponding collection time (Supplementary Table 4), suggesting hypoxia of the patient.

## Discussion

In this study, we observed distinct differences on plasma DNA coverage in TSS regions among control subjects and mild and severe cases with COVID-19 infection. By deciphering the expression pattern in plasma DNA based on TSS coverage profile, we also identified a series of up- and downregulated genes from the plasma expression pool of severe patient compared with mild patient. Further pathway and function analysis of these genes suggested their involvement of the COVID-19 progression.

In addition, we investigated the dysregulated genes in the blood cells of COVID-19 patient and lung cells with SARS-CoV-2 infection to trace the tissue origin of expression pattern in the plasma. We found interestingly that the pathways related to viral and antiviral activities identified in the plasma of severe patient were both enhanced in the blood cells of COVID-19 patients, indicating the viral replication was active in blood cells and the immune system of blood cells were intensively involved in the viral defense. Meanwhile, the genes identified in plasma DNA consistently downregulated in lung cells with SARS-CoV-2 infection were predominantly enriched in the ATP synthesis pathways, suggesting the decreased ATP and energy reserves due to the lung injury during SARS-COV-2 infection, which has been evidenced to be closely related to the severe COVID-19 symptoms (Patel and Sriram, 2009; van Kempen and Deixler, 2020). These findings were further clearly observed in another sample set. Therefore, the TSS coverage profile of these lung-specific genes could be targeted as potential markers to screen the patients with a high risk to develop severe symptoms at early stage.

Furthermore, we observed changes in mitochondrial cfDNA concentration, which matches with the hematocrit and hemoglobin of the patient.

A limitation of this study is the relatively small number of samples, which might have enhanced the possible influence of individual preferences. Thus, through the analysis of another independent set of samples, the observed characteristic patterns of TSS coverages and gene-specific expressions in severe patients were able to be consolidated, which enhanced the conclusions we drew from the former samples. We anticipate that further expanding the sample size and increasing the sequencing depth would allow us to deeply interpret the mechanisms underlying the disease severity and fully elaborate the capacity of this approach in the prediction of severe cases from patients with COVID-19.

In summary, the comprehensive analysis of TSS coverage profiles in mild and severe patients allowed us to discern the alteration of biological process caused by SARS-COV-2 infection. This study also demonstrated the utility of cfDNA in the discrimination of the severe patient from mild patients, as well as the surveillance, medication guidance, and prognosis of COVID-19 patients by targeting the TSS regions of the informative genes in a simple, fast, and low-cost manner.

## Data Availability Statement

The data were deposited in CNSA (CNGB Nucleotide Sequence Archive) of CNGBdb (China National GeneBank database) (https://db.cngb.org/cnsa/) and accession number: CNP0001059.

## Ethics Statement

The studies involving human participants were reviewed and approved by Medical Ethics Committee of Hainan General Hospital, Medical Ethics Committee of Union Hospital, Tongji Medical College, Huazhong University of Science and Technology, and the Institute Review Board of BGI. The patients/participants provided their written informed consent to participate in this study.

## Author Contributions

XJ, HZ, XC, HJ, SF, FJC, and RC designed this study. HZ, LL, YL, JX, FJC, YL, TK, and YT analyzed and interpreted the data. HZ, RC, and XC wrote the manuscript. XJ, FC, XX, KS, HY, and JianW revised the draft and provided important intellectual content. TW, ZM, HL, CZ, JiaoW, XL, FL, JH, ML, JZ, FC, CG, CH, JY, RO, FZ, and YJ performed sequencing and analyzed clinical data. All authors read and approved the manuscript.

## Conflict of Interest

The authors declare that the research was conducted in the absence of any commercial or financial relationships that could be construed as a potential conflict of interest.

## Supplementary Material

Supplementary Figure 1 Illustration of timelines for the mild and severe cases.

Supplementary Figure 2 Summary of identified up- **(A)** and downregulated **(B)** genes in plasma of severe patient from HN sample set, blood cells of COVID-19 patients, and lung cells with SARS-CoV-2 infection.

Supplementary Figure 3 Summary of identified up- **(A)** and downregulated **(B)** genes in plasma of severe patients from WH sample set, blood cells of COVID-19 patients, and lung cells with SARS-CoV-2 infection.

Supplementary Figure 4 Enriched pathways of genes with consistent alteration of gene expressions in both plasma and tissues in the WH sample set. Enriched pathways of genes with consistent down- **(A)** and upregulated pattern **(B)** in plasma of severe patient and blood cells of COVID-19 patients. Enriched pathways of genes with consistent down- **(C)** and upregulated pattern **(D)** in plasma of severe patient and lung cells of SARS-CoV-2 infection.

Supplementary Table 1 Genes with significantly different TSS coverage profiles in severe COVID-19 patient compared with mild patient.

Supplementary Table 2 Genes with significantly different expression levels in blood cells of COVID-19 patient and lung cells with SARS-CoV-2 infection compared with COVID-19-negative cases showed consistently altered pattern in plasma of our severe patient.

Supplementary Table 3 Microbiomes infected in plasma of cases and controls.

Supplementary Table 4 Clinical laboratory records of the mild and severe cases.

## References

[B1] Abu-Farha M. Thanaraj T. A. Qaddoumi M. G. Hashem A. Abubaker J. Al-Mulla F. (2020). The role of lipid metabolism in COVID-19 virus infection and as a drug target. *Int. J. Mol. Sci.* 21:3544. 10.3390/ijms21103544 32429572 PMC7278986

[B2] Bagam P. Singh D. P. Inda M. E. Batra S. (2017). Unraveling the role of membrane microdomains during microbial infections. *Cell Biol. Toxicol.* 33 429–455. 10.1007/s10565-017-9386-9 28275881 PMC7088210

[B3] Barnes B. J. Adrover J. M. Baxter-Stoltzfus A. Borczuk A. Cools-Lartigue J. Crawford J. M. (2020). Targeting potential drivers of COVID-19: neutrophil extracellular traps. *J. Exp. Med.* 217:e20200652. 10.1084/jem.20200652 32302401 PMC7161085

[B4] Barrett T. Wilhite S. E. Ledoux P. Evangelista C. Kim I. F. Tomashevsky M. (2013). NCBI GEO: archive for functional genomics data sets–update. *Nucleic Acids Res.* 41 D991–D995. 10.1093/nar/gks1193 23193258 PMC3531084

[B5] Blanco-Melo D. Nilsson-Payant B. E. Liu W. C. Uhl S. Hoagland D. Møller R. (2020). Imbalanced host response to SARS-CoV-2 drives development of COVID-19. *Cell* 181 1036–1045.e9. 10.1016/j.cell.2020.04.026 32416070 PMC7227586

[B6] Chen Y. Chen Y. Shi C. Huang Z. Zhang Y. Li S. (2018). SOAPnuke: a MapReduce acceleration-supported software for integrated quality control and preprocessing of high-throughput sequencing data. *Gigascience* 7 1–6. 10.1093/gigascience/gix120 29220494 PMC5788068

[B7] Cheng A. P. Cheng M. P. Gu W. Lenz J. S. Hsu E. Schurr E. (2020). Cell-free DNA in blood reveals significant cell, tissue and organ specific injury and predicts COVID-19 severity. *medRxiv* [Preprint] 10.1101/2020.07.27.20163188 32766608 PMC7402071

[B8] Chukkapalli V. Heaton N. S. Randall G. (2012). Lipids at the interface of virus-host interactions. *Curr. Opin. Microbiol.* 15 512–518. 10.1016/j.mib.2012.05.013 22682978 PMC3424344

[B9] Cizmecioglu A. Cizmecioglu H. A. Goktepe M. H. Emsen A. Korkmaz C. Tasbent F. E. (2020). Apoptosis−induced T cell lymphopenia is related to COVID−19 severity. *J. Med. Virol.* 93 2867–2874. 10.1002/jmv.26742 33331657

[B10] Cooper K. W. Brann D. H. Farruggia M. C. Bhutani S. Pellegrino R. Tsukahara T. (2020). COVID-19 and the chemical senses: supporting players take center stage. *Neuron* 107 219–233. 32640192 10.1016/j.neuron.2020.06.032 PMC7328585

[B11] Diamond D. L. Syder A. J. Jacobs J. M. Sorensen C. M. Walters K. A. Proll S. C. (2010). Temporal proteome and lipidome profiles reveal hepatitis C virus-associated reprogramming of hepatocellular metabolism and bioenergetics. *PLoS Pathog.* 6:e1000719. 10.1371/journal.ppat.1000719 20062526 PMC2796172

[B12] Galougahi M. K. Ghorbani J. Bakhshayeshkaram M. Naeini A. S. Haseli S. (2020). Olfactory bulb magnetic resonance imaging in SARS-CoV-2-induced anosmia: the first report. *Acad. Radiol.* 27 892–893. 10.1016/j.acra.2020.04.002 32295727 PMC7151240

[B13] Gardinassi L. G. Souza C. O. S. Sales-Campos H. Fonseca S. G. (2020). Immune and metabolic signatures of COVID-19 revealed by transcriptomics data reuse. *Front. Immunol.* 11:1636. 10.3389/fimmu.2020.01636 32670298 PMC7332781

[B14] Gill S. E. dos Santos C. C. O’Gorman D. B. Carter D. E. Patterson E. K. Slessarev M. (2020). Transcriptional profiling of leukocytes in critically ill COVID19 patients: implications for interferon response and coagulation. *Intensive Care Med. Exp.* 8:75. 10.1186/s40635-020-00361-9 33306162 PMC7729690

[B15] Gordon D. E. Jang G. M. Bouhaddou M. Xu J. Obernier K. White K. M. (2020). A SARS-CoV-2 protein interaction map reveals targets for drug repurposing. *Nature* 583 459–468. 10.1038/s41586-020-2286-9 32353859 PMC7431030

[B16] Gralinski L. E. Sheahan T. P. Morrison T. E. Menachery V. D. Jensen K. Leist S. R. (2018). Complement activation contributes to severe acute respiratory syndrome coronavirus pathogenesis. *mBio* 9:e01753-18. 10.1128/mBio.01753-18 30301856 PMC6178621

[B17] Grasselli G. Zangrillo A. Zanella A. Antonelli M. Cabrini L. Castelli A. (2020). Baseline characteristics and outcomes of 1591 patients infected with SARS-CoV-2 admitted to ICUs of the lombardy region, Italy. *JAMA J. Am. Med. Assoc.* 323 1574–1581. 10.1001/jama.2020.5394 32250385 PMC7136855

[B18] Hsu N. Y. Ilnytska O. Belov G. Santiana M. Chen Y. H. Takvorian P. M. (2010). Viral reorganization of the secretory pathway generates distinct organelles for RNA replication. *Cell* 141 799–811. 10.1016/j.cell.2010.03.050 20510927 PMC2982146

[B19] Iyer S. S. Pulskens W. P. Sadler J. J. Butter L. M. Teske G. J. Ulland T. K. (2009). Necrotic cells trigger a sterile inflammatory response through the Nlrp3 inflammasome. *Proc. Natl. Acad. Sci. U.S.A.* 106 20388–20393. 10.1073/pnas.0908698106 19918053 PMC2787135

[B20] Kendig K. I. Baheti S. Bockol M. A. Drucker T. M. Hart S. N. Heldenbrand J. R. (2019). SentIeon DNaSeq variant calling workflow demonstrates strong computational performance and accuracy. *Front. Genet.* 10:736. 10.3389/fgene.2019.00736 31481971 PMC6710408

[B21] Kouhpayeh S. Shariati L. Boshtam M. Rahimmanesh I. Mirian M. Zeinalian M. (2020). The molecular story of COVID-19; NAD+ Depletion addresses all questions in this infection. *Preprints* 10.20944/preprints202003.0346.v1 32283112

[B22] Le T. T. T. Berg N. K. Harting M. T. Li X. Eltzschig H. K. Yuan X. (2019). Purinergic signaling in pulmonary inflammation. *Front. Immunol.* 10:1633. 10.3389/fimmu.2019.01633 31379836 PMC6646739

[B23] Lechien J. R. Chiesa-Estomba C. M. De Siati D. R. Horoi M. Le Bon S. D. Rodriguez A. (2020). Olfactory and gustatory dysfunctions as a clinical presentation of mild-to-moderate forms of the coronavirus disease (COVID-19): a multicenter European study. *Eur. Arch. Oto Rhino Laryngol.* 277 2251–2261. 10.1007/s00405-020-05965-1 32253535 PMC7134551

[B24] Li H. Durbin R. (2010). Fast and accurate long-read alignment with burrows-wheeler transform. *Bioinformatics* 26 589–595. 10.1093/bioinformatics/btp698 20080505 PMC2828108

[B25] Love M. I. Huber W. Anders S. (2014). Moderated estimation of fold change and dispersion for RNA-seq data with DESeq2. *Genome Biol.* 15:550. 10.1186/s13059-014-0550-8 25516281 PMC4302049

[B26] Mankouri J. Tedbury P. R. Gretton S. Hughes M. E. Griffin S. D. C. Dallas M. L. (2010). Enhanced hepatitis C virus genome replication and lipid accumulation mediated by inhibition of AMP-activated protein kinase. *Proc. Natl. Acad. Sci. U.S.A.* 107 11549–11554. 10.1073/pnas.0912426107 20534540 PMC2895084

[B27] McKenna A. Hanna M. Banks E. Sivachenko A. Cibulskis K. Kernytsky A. (2010). The genome analysis toolkit: a MapReduce framework for analyzing next-generation DNA sequencing data. *Genome Res.* 20 1297–1303. 10.1101/gr.107524.110 20644199 PMC2928508

[B28] Menni C. Valdes A. M. Freidin M. B. Sudre C. H. Nguyen L. H. Drew D. A. (2020). Real-time tracking of self-reported symptoms to predict potential COVID-19. *Nat. Med.* 26 1037–1040. 10.1038/s41591-020-0916-2 32393804 PMC7751267

[B29] Nagy P. D. Strating J. R. P. M. van Kuppeveld F. J. M. (2016). Building viral replication organelles: close encounters of the membrane types. *PLoS Pathog.* 12:e1005912. 10.1371/journal.ppat.1005912 27788266 PMC5082816

[B30] Nomura J. So A. Tamura M. Busso N. (2015). Intracellular ATP decrease mediates NLRP3 inflammasome activation upon nigericin and crystal stimulation. *J. Immunol.* 195 5718–5724. 10.4049/jimmunol.1402512 26546608

[B31] Parma V. Ohla K. Veldhuizen M. G. Niv M. Y. Kelly C. E. Bakke A. J. (2020). More than smell. COVID-19 is associated with severe impairment of smell, taste, and chemesthesis. *medRxiv* [Preprint] 10.1101/2020.05.04.20090902 PMC7337664 32564071

[B32] Patel U. Sriram K. (2009). Acute respiratory failure due to refeeding syndrome and hypophosphatemia induced by hypocaloric enteral nutrition. *Nutrition* 25 364–367. 10.1016/j.nut.2008.09.011 19062257

[B33] Phua J. Weng L. Ling L. Egi M. Lim C. M. Divatia J. V. (2020). Intensive care management of coronavirus disease 2019 (COVID-19): challenges and recommendations. *Lancet Respir. Med.* 8 506–517. 10.1016/S2213-2600(20)30161-2 32272080 PMC7198848

[B34] Richardson S. Hirsch J. S. Narasimhan M. Crawford J. M. McGinn T. Davidson K. W. (2020). Presenting characteristics, comorbidities, and outcomes among 5700 patients hospitalized with COVID-19 in the New York City area. *JAMA J. Am. Med. Assoc.* 323 2052–2059. 10.1001/jama.2020.6775 32320003 PMC7177629

[B35] Schones D. E. Cui K. Cuddapah S. Roh T. Y. Barski A. Wang Z. (2008). Dynamic regulation of nucleosome positioning in the human genome. *Cell* 132 887–898. 10.1016/j.cell.2008.02.022 18329373 PMC10894452

[B36] Snyder M. W. Kircher M. Hill A. J. Daza R. M. Shendure J. (2016). Cell-free DNA comprises an in vivo nucleosome footprint that informs its tissues-of-origin. *Cell* 164 57–68. 10.1016/j.cell.2015.11.050 26771485 PMC4715266

[B37] Sorokin A. V. Karathanasis S. K. Yang Z. H. Freeman L. Kotani K. Remaley A. T. (2020). COVID-19—associated dyslipidemia: implications for mechanism of impaired resolution and novel therapeutic approaches. *FASEB J.* 34 9843–9853. 10.1096/fj.202001451 32588493 PMC7361619

[B38] Sun K. Jiang P. Chan K. C. A. Wong J. Cheng Y. K. Y. Y. Liang R. H. S. S. (2015). Plasma DNA tissue mapping by genome-wide methylation sequencing for noninvasive prenatal, cancer, and transplantation assessments. *Proc. Natl. Acad. Sci. U.S.A.* 112 E5503–E5512. 10.1073/pnas.1508736112 26392541 PMC4603482

[B39] Sun K. Jiang P. Cheng S. H. Cheng T. H. T. Wong J. Wong V. W. S. (2019). Orientation-aware plasma cell-free DNA fragmentation analysis in open chromatin regions informs tissue of origin. *Genome Res.* 29 418–427. 10.1101/gr.242719.118 30808726 PMC6396422

[B40] Sun K. Jiang P. Wong A. I. C. Cheng Y. K. Y. Cheng S. H. Zhang H. (2018). Size-tagged preferred ends in maternal plasma DNA shed light on the production mechanism and show utility in noninvasive prenatal testing. *Proc. Natl. Acad. Sci. U.S.A.* 115 E5106–E5114. 10.1073/pnas.1804134115 29760053 PMC5984542

[B41] Taube S. Jiang M. Wobus C. E. (2010). Glycosphingolipids as receptors for non-enveloped viruses. *Viruses* 2 1011–1049. 10.3390/v2041011 21994669 PMC3185660

[B42] Taylor G. H. (2003). Cytomegalovirus. *Am. Fam. Physician.* 67 519–524. 12588074

[B43] Thierry A. R. Roch B. (2020). Neutrophil extracellular traps and by-products play a key role in COVID-19: pathogenesis, risk factors, and therapy. *J. Clin. Med.* 9:2942. 10.3390/jcm9092942 32933031 PMC7565044

[B44] Tian H. Liu Y. Li Y. Wu C. H. Chen B. Kraemer M. U. G. (2020). An investigation of transmission control measures during the first 50 days of the COVID-19 epidemic in China. *Science* 368 638–642. 10.1126/science.abb6105 32234804 PMC7164389

[B45] Trautmann A. (2009). Extracellular ATP in the immune system: more than just a “danger signal.” *Sci. Signal.* 2:e6. 10.1126/scisignal.256pe6 19193605

[B46] Ulz P. Thallinger G. G. Auer M. Graf R. Kashofer K. Jahn S. W. (2016). Inferring expressed genes by whole-genome sequencing of plasma DNA. *Nat. Genet.* 48 1273–1278. 10.1101/049478 27571261

[B47] van Kempen T. A. Deixler E. (2020). SARS-CoV-2: influence of phosphate and magnesium, moderated by vitamin D, on energy (ATP)-metabolism and on severity of COVID-19. *Am. J. Physiol. Metab.* 320 E2–E6. 10.1152/ajpendo.00474.2020 33174766 PMC7816430

[B48] Venkatesh S. Workman J. L. (2015). Histone exchange, chromatin structure and the regulation of transcription. *Nat. Rev. Mol. Cell Biol.* 16 178–189. 10.1038/nrm3941 25650798

[B49] Vincent J. L. Taccone F. S. (2020). Understanding pathways to death in patients with COVID-19. *Lancet Respir. Med.* 8 430–432. 10.1016/S2213-2600(20)30165-X 32272081 PMC7270480

[B50] V’kovski P. Gerber M. Kelly J. Pfaender S. Ebert N. Braga Lagache S. (2019). Determination of host proteins composing the microenvironment of coronavirus replicase complexes by proximity-labeling. *Elife* 8:e42037. 10.7554/eLife.42037 30632963 PMC6372286

[B51] WHO (2020). *Laboratory Biosafety Guidance Related to Coronavirus Disease 2019 (COVID-19). Interim Guidance.* Geneva: World Health Organization.

[B52] World Health Organization (2021). *COVID-19 Weekly Epidemiological Update 22.* Geneva: World Health Organization, 1–3.

[B53] Wu D. Shu T. Yang X. Song J.-X. Zhang M. Yao C. (2020). Plasma metabolomic and lipidomic alterations associated with COVID-19. *Natl. Sci. Rev.* 7, 1157–1168. 10.1093/nsr/nwaa086 PMC7197563 34676128

[B54] Wu Z. McGoogan J. M. (2020). Characteristics of and important lessons from the coronavirus disease 2019 (COVID-19) outbreak in China. *JAMA* 323 1239–1242. 10.1001/jama.2020.2648 32091533

[B55] Young B. E. Ong S. W. X. Kalimuddin S. Low J. G. Tan S. Y. Loh J. (2020). Epidemiologic features and clinical course of patients infected with SARS-CoV-2 in Singapore. *JAMA J. Am. Med. Assoc.* 323 1488–1494. 10.1001/jama.2020.3204 32125362 PMC7054855

[B56] Yu G. Wang L. G. Han Y. He Q. Y. (2012). ClusterProfiler: an R package for comparing biological themes among gene clusters. *Omi. A J. Integr. Biol.* 16 284–287. 10.1089/omi.2011.0118 22455463 PMC3339379

[B57] Zhou F. Yu T. Du R. Fan G. Liu Y. Liu Z. (2020). Clinical course and risk factors for mortality of adult inpatients with COVID-19 in Wuhan, China: a retrospective cohort study. *Lancet* 395 1054–1062. 10.1016/S0140-6736(20)30566-3 32171076 PMC7270627

[B58] Zhou P. Yang X. L. Wang X. G. Hu B. Zhang L. Zhang W. (2020). A pneumonia outbreak associated with a new coronavirus of probable bat origin. *Nature* 588:E6. 10.1038/s41586-020-2012-7 33199918 PMC9744119

[B59] Zhou Y. Zhou B. Pache L. Chang M. Khodabakhshi A. H. Tanaseichuk O. (2019). Metascape provides a biologist-oriented resource for the analysis of systems-level datasets. *Nat. Commun.* 10:1523. 10.1038/s41467-019-09234-6 30944313 PMC6447622

[B60] Zhu N. Zhang D. Wang W. Li X. Yang B. Song J. (2020). A novel coronavirus from patients with pneumonia in China, 2019. *N. Engl. J. Med.* 382 727–733. 10.1056/nejmoa2001017 31978945 PMC7092803

[B61] Zuo Y. Yalavarthi S. Shi H. Gockman K. Zuo M. Madison J. A. (2020). Neutrophil extracellular traps in COVID-19. *JCI Insight* 5:e138999. 10.1172/jci.insight.138999 32329756 PMC7308057

