# Supplementary figures and images for: Transcriptional Start Site Coverage Analysis in Plasma Cell-Free DNA Reveals Disease Severity and Tissue Specificity of COVID-19 Patients
Source: Front Genet. 2021 May 28;12:663098. doi: 10.3389/fgene.2021.663098 (PMC8194351; doi:10.3389/fgene.2021.663098)

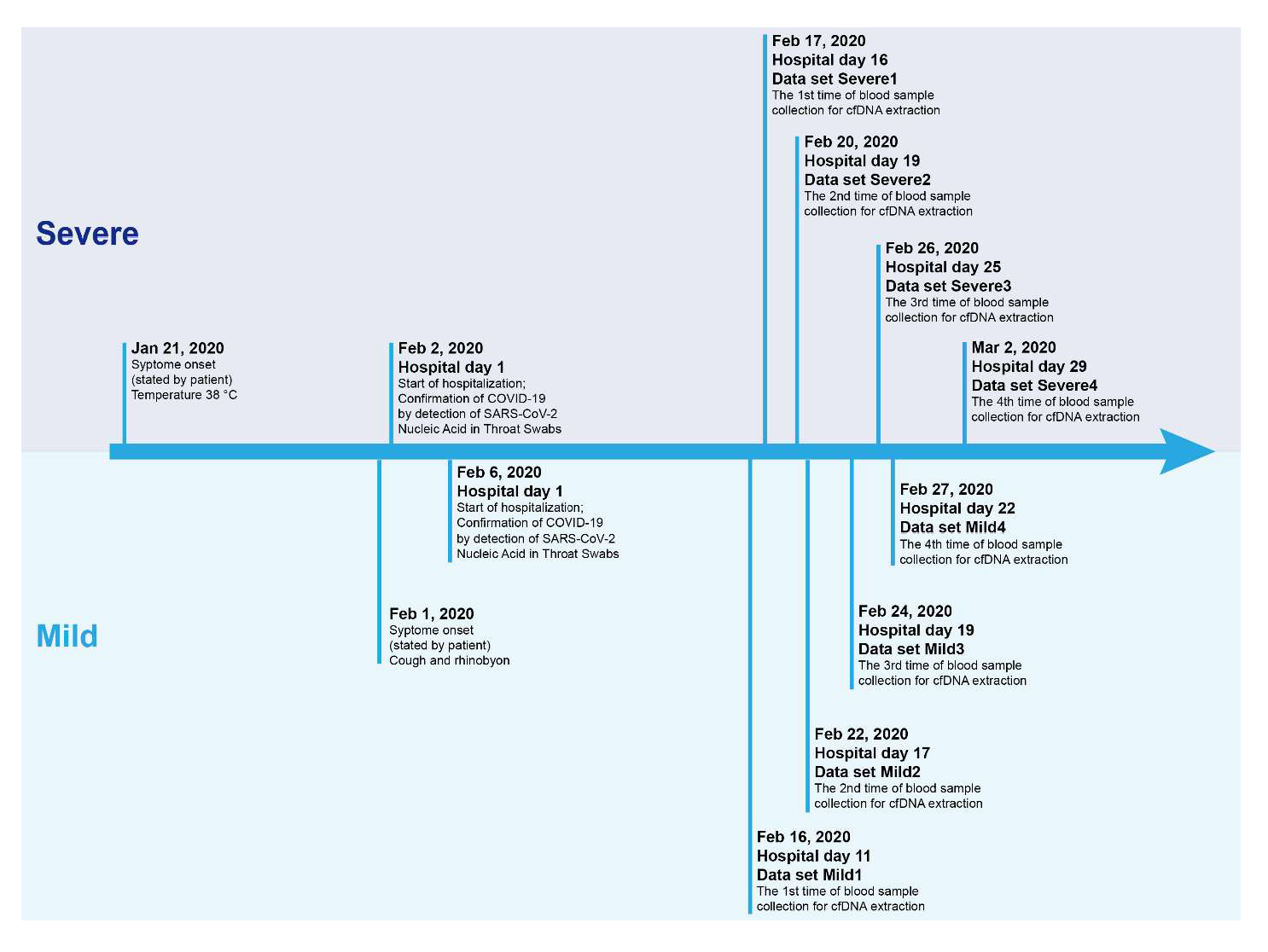

Supplement: Supplementary Figure 1 — Illustration of timelines for the mild and severe cases. [file Image_1.TIFF]

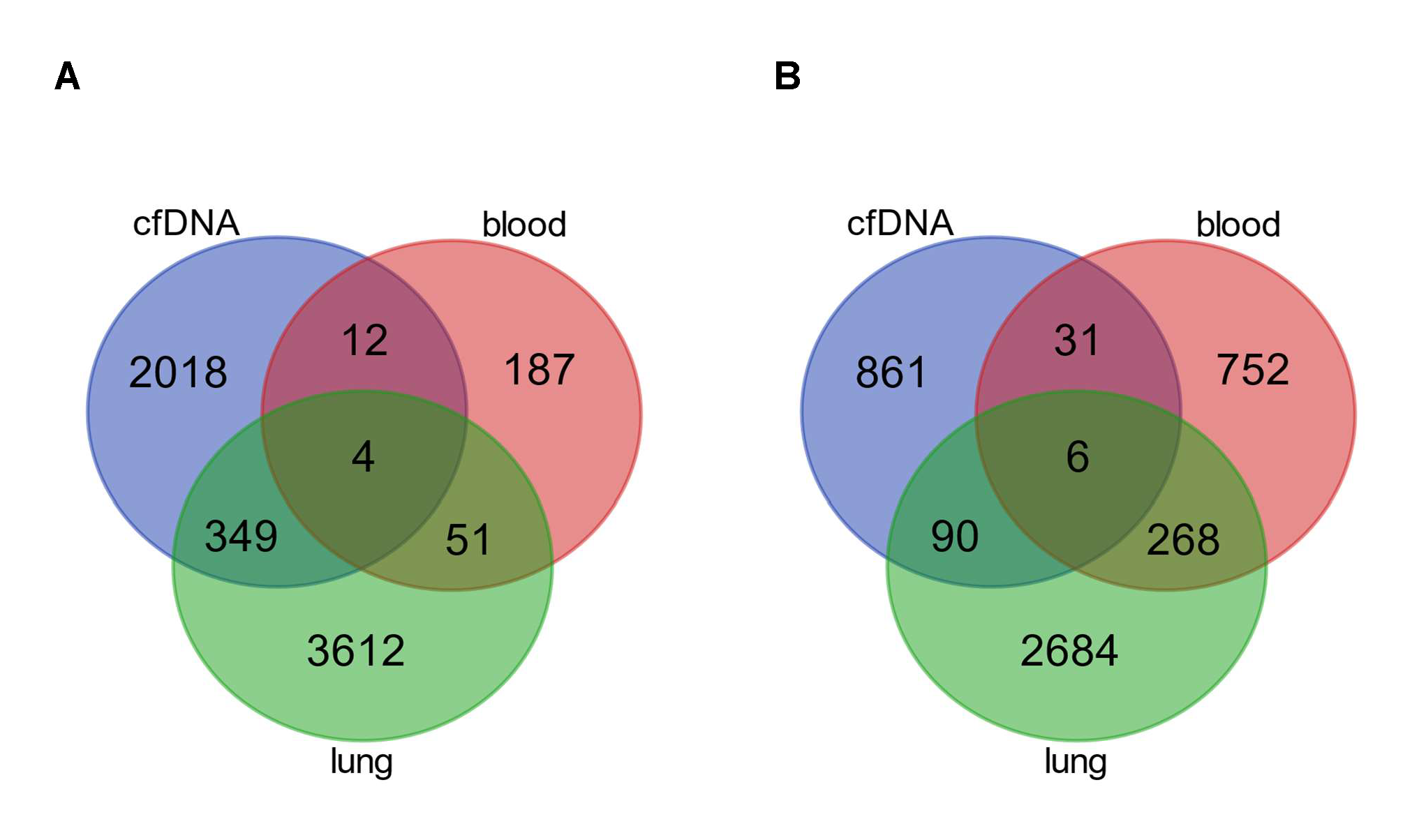

Supplement: Supplementary Figure 2 — Summary of identified up- (A) and downregulated (B) genes in plasma of severe patient from HN sample set, blood cells of COVID-19 patients, and lung cells with SARS-CoV-2 infection. [file Image_2.TIFF]

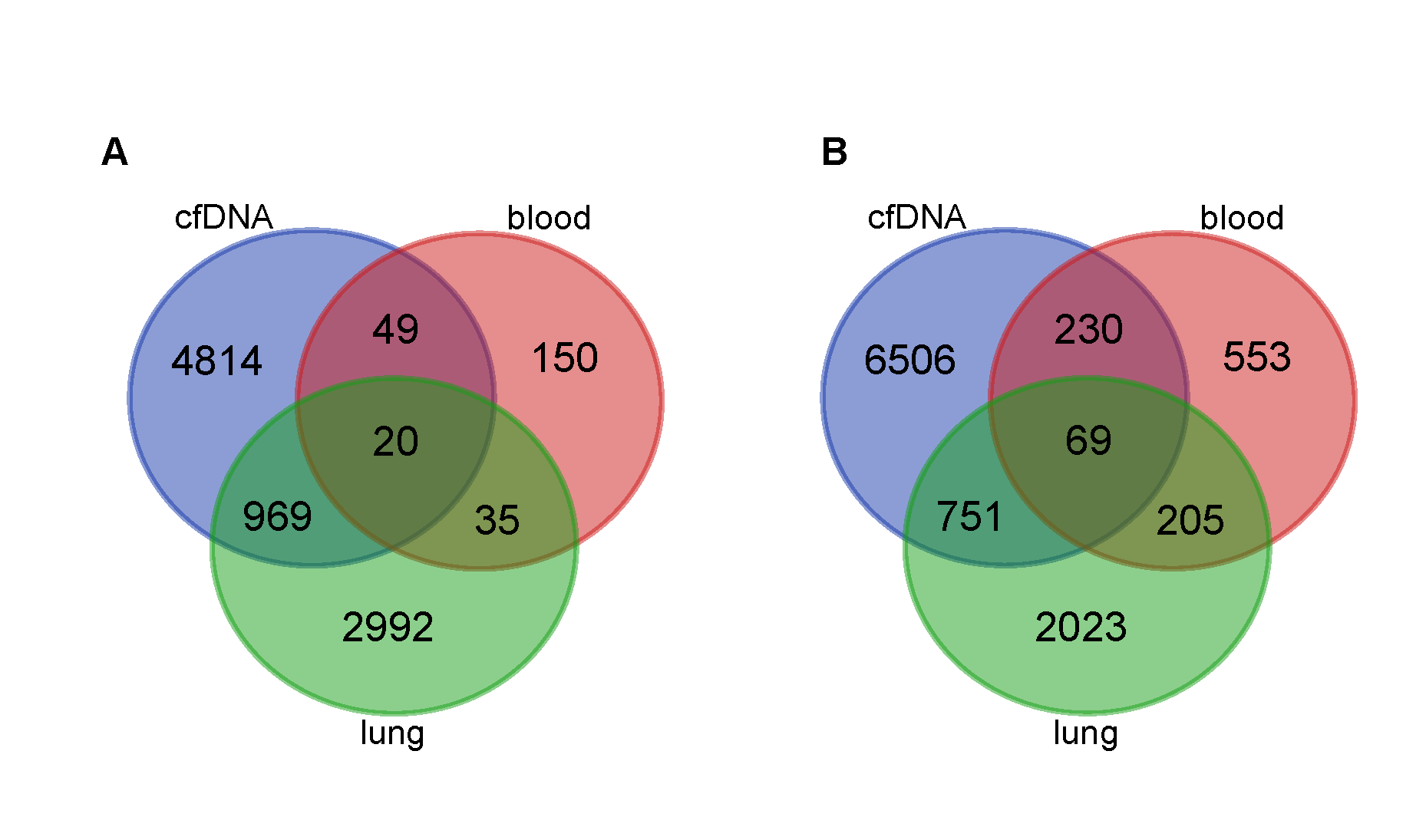

Supplement: Supplementary Figure 3 — Summary of identified up- (A) and downregulated (B) genes in plasma of severe patients from WH sample set, blood cells of COVID-19 patients, and lung cells with SARS-CoV-2 infection. [file Image_3.TIFF]

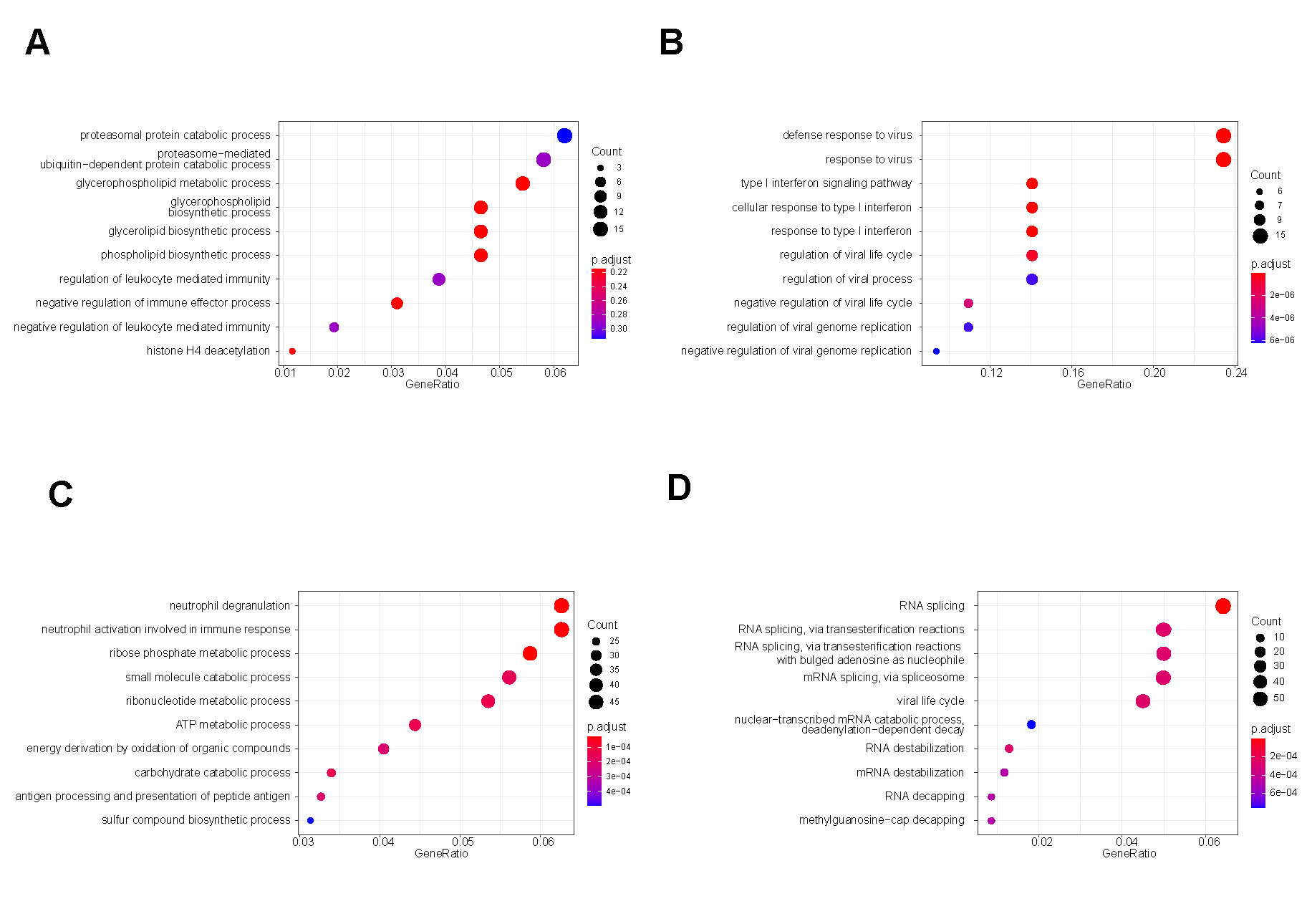

Supplement: Supplementary Figure 4 — Enriched pathways of genes with consistent alteration of gene expressions in both plasma and tissues in the WH sample set. Enriched pathways of genes with consistent down- (A) and upregulated pattern (B) in plasma of severe patient and blood cells of COVID-19 patients. Enriched pathways of genes with consistent down- (C) and upregulated pattern (D) in plasma of severe patient and lung cells of SARS-CoV-2 infection. [file Image_4.TIFF]
